# Did the 2008 Financial Crisis (Literally) Change Minds?

**DOI:** 10.1093/geroni/igaa057.201

**Published:** 2020-12-16

**Authors:** Cliff Whetung, Abhinav Bhattacharyya

**Affiliations:** 1 NYU, New York, New York, United States; 2 New York University, New York, New York, United States

## Abstract

This paper investigated whether experiencing a major asset shock (30% or greater decrease in total assets) changed the cognitive health trajectories of older adults (aged 65+ at baseline)? There is a robust literature supporting a relationship between potentially modifiable environmental and individual risk factors and cognitive health and emerging literature supporting a relationship between physical health shocks and later-life financial stability in both the US and internationally. This analysis employed six waves of Health and Retirement Study Core Data (2006-2014) to estimate the causal effect of major asset losses in the period of the 2008 financial crisis. We matched respondents using a rich variety of covariates including education, race/ethnicity, income, total assets, chronic health conditions, depressive symptoms, frequency of physical activity engagement, and engagement with religious activities. Using both propensity score-matched growth curve and difference-in-differences models, we found a small, but significant, negative relationship between major asset shocks and total cognition scores. We also identified a significant overall negative effect on total cognition on all older adults in this sample regardless of exposure to asset shocks. Additionally, both the frequency of asset shock exposure and magnitude of effect on total cognition scores was larger for low-income African-American and Hispanic older adults. These findings suggest in part that recovery from large economic crises may be especially difficult for older adults occupying vulnerable socioeconomic positions and that such events may accelerate the cognitive decline of those who are at or near retirement when asset shocks occur.

